# The Impact of Conventional Chemotherapy Regimens and Targeted Drugs on Ovarian Function in Breast Cancer Patients

**DOI:** 10.1007/s43032-026-02067-x

**Published:** 2026-03-02

**Authors:** Seyma Haskoylu, Sevval Berfin Sahin, Alara Altıntas, Sule Yildiz, Gamze Bildik, Can Benlioglu, Volkan Turan, Samuel Kim, Ozgur Oktem

**Affiliations:** 1 Department of Obstetrics and Gynecology, Koç University Hospital, Koç University School of Medicine, Davutpasa Street No:4, Topkapi, Istanbul, 34010 Turkey; 2 Department of Experimental Therapeutics, The University of Texas MD Anderson Cancer Center, Houston, TX 77054 USA; 3 Translational Research Center, Koc University, Istanbul, Turkey; 4 Department of Obstetrics and Gynecology, Istanbul Health and Technology University, Istanbul, Turkey; 5 Eden Center for Advanced Fertility, Fullerton, CA USA

**Keywords:** Breast cancer, Ovarian reserve, Chemotherapy, PARP inhibitors, Immune check point inhibitors, Cyclin dependent kinase inhibitors, Amenorrhea, Ovarian function

## Abstract

Breast cancer is the most common malignancy among women, affecting nearly 1.5 million individuals worldwide every year. While survival rates improve, many reproductive-age survivors confront significant long-term consequences, particularly diminished ovarian reserve, infertility, and premature ovarian insufficiency due to the gonadotoxic effects of chemotherapy. Postponement of pregnancy for five years or more after treatment exacerbates the decline in fertility due to ongoing ovarian aging and depletion of residual ovarian reserve. Women carrying BRCA1/2 mutations may already exhibit reduced ovarian reserve and are more vulnerable to gonadal damage, possibly due to impaired DNA repair mechanisms associated with these mutations. The contribution of other breast cancer susceptibility genes (e.g., ATM, CHEK2, PALB2, BARD1, RAD51C, RAD51D, and TP53) to chemotherapy-induced gonadotoxicity remains unclear. Although animal data shows depletion of primordial follicle pool and granulosa cells dysfunction, the ovarian effects of the poly(ADP-ribose) polymerase (PARP) inhibitors in women with and without BRCA mutation are not clear. Immune-check point inhibitors (ICIs) causes immune-mediated destruction of the primordial follicle pool and reduction in ovarian reserve. Cyclin dependent kinase inhibitors appear to be less toxic than ICIs. In this narrative review of the current literature we aimed to provide a comprehensive overview of the molecular mechanisms underlying ovarian toxicity associated with conventional chemotherapy and targeted therapies in breast cancer treatment.

## Introduction

Breast cancer is the most common malignancy in women of reproductive age [1]. The 5-year relative survival rate has increased to 99% among women with localized breast cancer [2]. Exposure to chest radiation for Hodgkin lymphoma during childhood increases the risk of breast cancer in adulthood [3]. Increased life expectancy has brought the concerns about fertility as a quality of life issue among young survivors of breast cancer who have not completed childbearing because many of them develop amenorrhea, infertility or premature ovarian insufficiency (POI) after exposure to adjuvant chemotherapy regimens [4]. Moreover, postponement of pregnancy for years will further increase the risk of infertility in breast cancer patients because of the natural decline of the ovarian reserve due to aging. During the initial visit, counseling about the risk of infertility and offering personalized risk assessment and the options to preserve fertility are essential. While there is abundant data on the gonadotoxic effects of conventional combined chemotherapy regimens data is still limited regarding the ovarian effects of targeted therapies such as PARP inhibitors, Trastuzumab (anti-HER2/neu antibody), Lapatinib (anti-HER2/neu and EGFR antibody), immune check point inhibitor and cyclin-depdendent kinase 4/6 inhibitors. In this context, we aim to provide a comprehensive update by compartmantalizing the topic into the following sections:

1. Molecular mechanisms of ovarian damage for conventional chemotherapy regimens used in the treatment of breast cancer.
2. The risk of ovarian failure and amenorrhea after receiving conventional chemotherapy drugs.
3. The impact of targeted therapies on ovarian function.
4. The role of BRCA status and other breast cancer susceptibility genes in ovarian function and reserve before and after chemotherapy.

## Materials and Methods

We identified peer-reviewed articles and abstracts between 1985 and 2021 after searching PubMed as well as the proceedings of the professional society meetings relevant to cancer and reproductive medicine (American Society of Clinical Oncology, San Antonio Breast Cancer Symposium, European Cancer Conference, European Society of Medical Oncology, American Society for Reproductive Medicine, and European Society of Human Reproduction and Embryology).

## Results

### Molecular Mechanisms of Chemotherapy-related Ovarian Damage and Follicle Loss

**Fig. 1:**
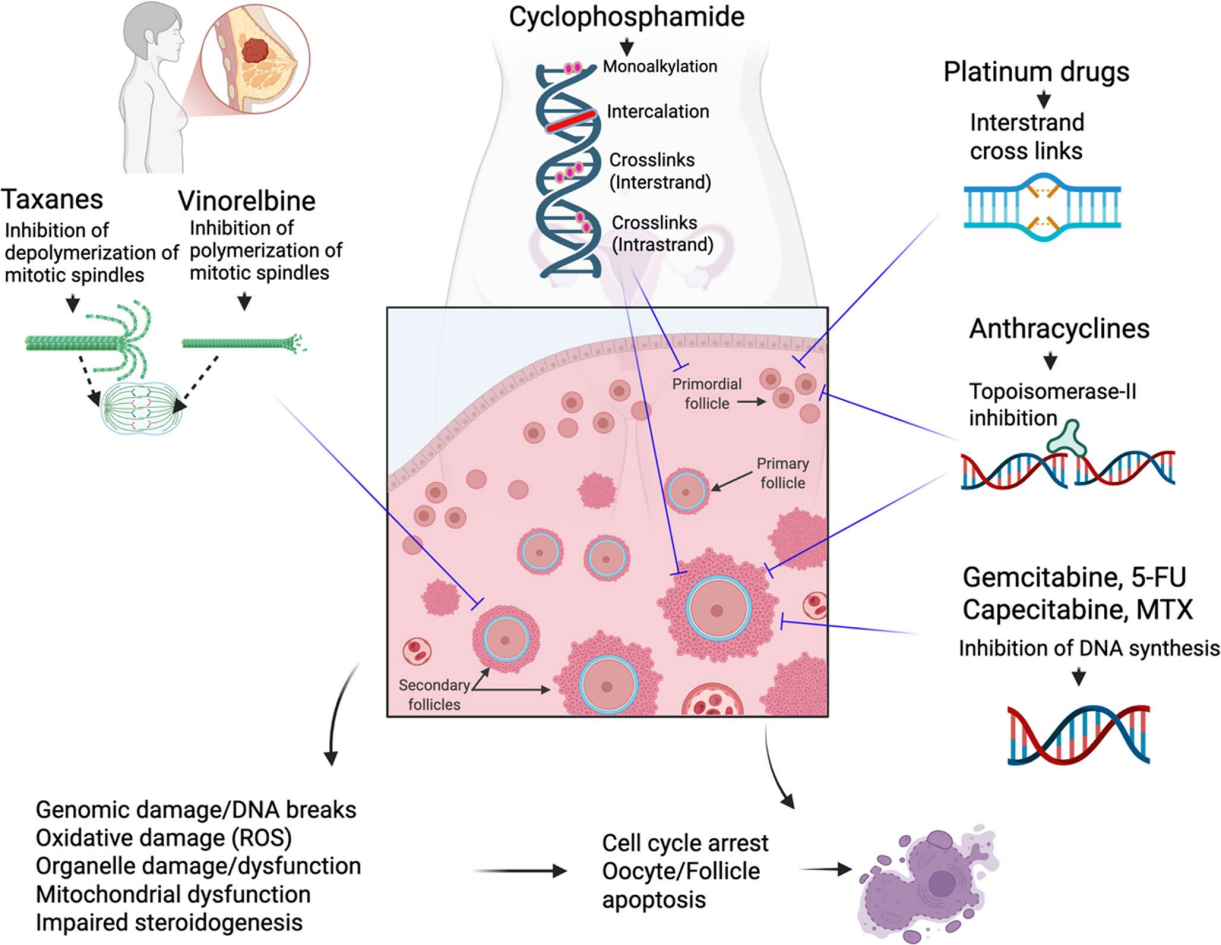
Conventional chemotherapy drugs used in the treatment of breast cancer and their mechanisms of action according to the cell cycle stage. Drugs that exert their anti-proliferative effects at a specific cell cycle stage, such as mitosis-specific taxanes and S-phase-specific antimetabolites, are less detrimental to the ovary than cell cycle–nonspecific alkylating agents, which cause more widespread ovarian destruction. *ROS*, reactive oxygen species; *5-FU*, 5-fluorouracil; *MTX*, methotrexate

**Fig. 2:**
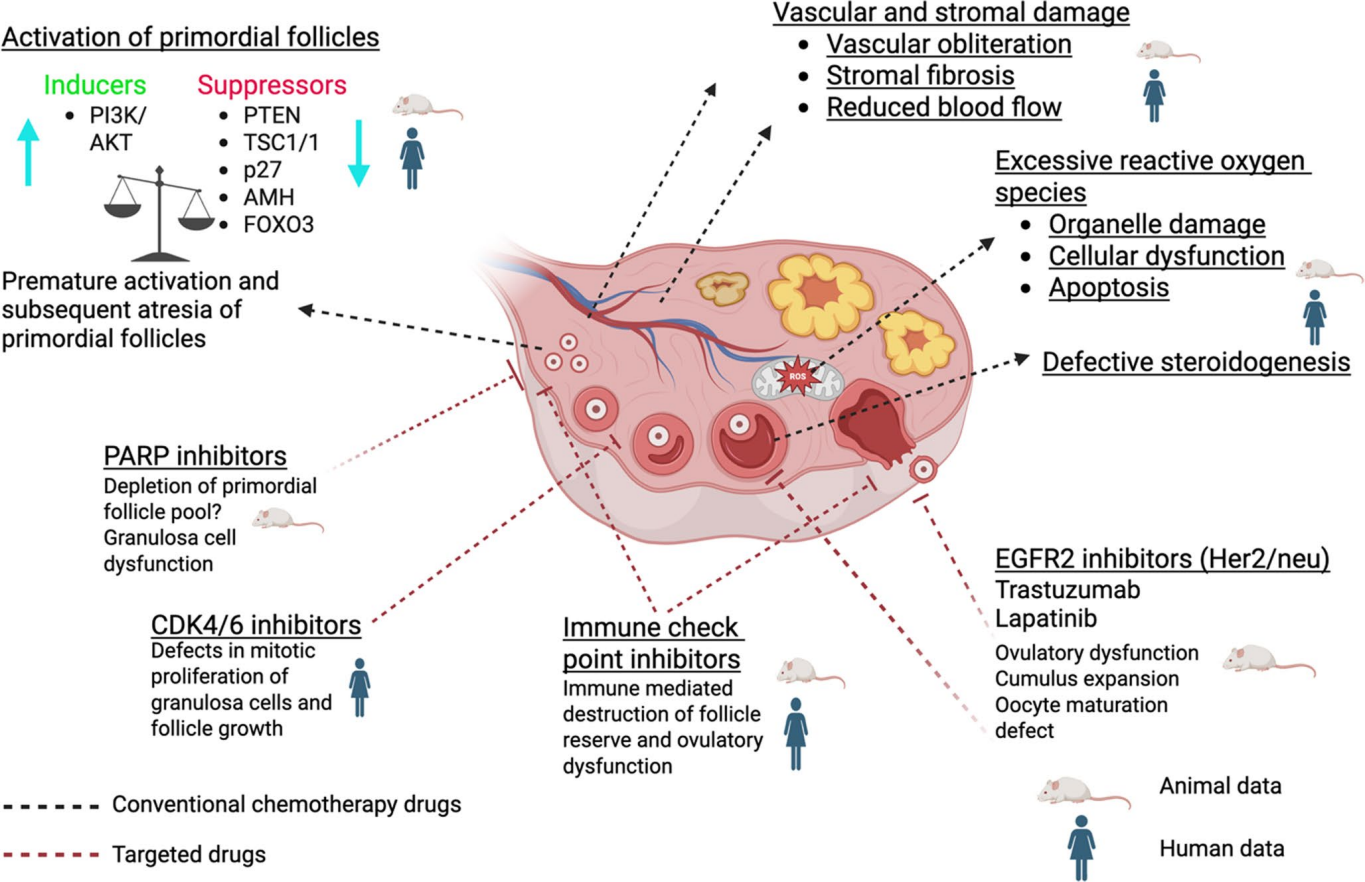
Overview of the proposed mechanisms by which conventional chemotherapy and targeted anticancer agents compromise ovarian function. The central illustration depicts a cross-section of a human ovary, demonstrating follicles at different stages. Black dashed arrows trace pathways known to be affected by conventional cytotoxic drugs, whereas red dashed arrows denote effects reported for targeted agents. Symbols depicting a rat indicate findings derived predominantly from animal models, while the human figure denotes mechanisms supported by human clinical, histological or translational data

The impact of chemotherapy agents on human ovary occurs via several different mechanisms First, they induce apoptotic death of oocyte and granulosa cells of primordials and growing follicles by exerting direct cytotoxic effects and/or causing additional harmful effects on ovarian stroma and vascular structures (Figs. 1 and 2) [5]. Among chemotherapy drugs, alkylating agents cause the most extensive ovarian damage and follicle destruction. Moreover, these agents induce genomic damage in somatic cells of dormant primordial and growing (secondary) follicles and cause massive follicle loss [5–8]. Second mechanism is the vascular and stromal damage related to chemotherapy-induced cytotoxicity. Certain chemotherapy drugs such as platinium group preferentially target vascular structures in the cortical and medullary portions of the human ovary, resulting in obliteration, fibrosis, and decreases in vascular endothelial growth factor (VEGF) and microvascular intensity [8–11]. The resultant decrease in ovarian vascularization may impair blood supply, aggravating follicle loss further and accelerating ovary aging. Disruption in ovarian vascular supply sometimes can be so severe that it can even be detected on doppler examination of ovarian vascularity as shown by a study in which young breast cancer patients treated with anthracyclines and taxanes. Ovarian blood flow was significantly reduced shortly following chemotherapy: Resistive index (RI) decreased by 52.5% and pulsatility index (PI) decreased by 24.2%. The mean ovarian size declined by 19.08% along with reduction in serum anti-Müllerian hormone levels [12].

The third mechanism of follicle loss is the “burn-out” phenomenon, characterized by the massive premature activation of dormant primordial follicles. Normally, the PI3K/Akt/mTOR signaling pathway, which drives follicle activation, is held in check by local inhibitory factors, including anti-Müllerian hormone (AMH) secreted by the growing follicle pool [13]. Chemotherapy-induced destruction of these anti-Müllerian hormone-producing growing follicles leads to a precipitous drop in local anti-Müllerian hormone levels. This reduction disinhibits the PI3K pathway, resulting in the downstream phosphorylation and nuclear export of the transcription factor FOXO3a. Consequently, the inhibitory clamp on the primordial follicles is released, triggering their massive entry into the growing pool and subsequent atresia due to the toxic environment [14]. Systemic administration of anti-Müllerian hormone attenuated cyclophosphamide-induced follicle loss by decreasing phosphorylation FOXO3A and possibly inhibiting PI3K pathway in mouse ovary [15]. It is important to note that this mechanism has been primarily established in rodent models, particularly with cyclophosphamide. Evidence in humans remains limited and controversial. While some in vitro human ovarian cortical studies support PI3K pathway upregulation following chemotherapy exposure, in vivo human ovarian xenograft studies suggest that direct DNA damage (apoptosis) rather than “burn-out” is the dominant mechanism of primordial follicle loss in humans [5, 16].

The forth proposed mechanism, at least conducted with mice ovaries, explains the different mechanisms of follicle loss. For example, while cisplatin exposure causes oocyte-specific damage, doxorubicin induces more selective damage to the mitotic granulosa cells of secondary follicles [14].

As an index drug of alkylating category cyclophosphamide is included in several combined regimens (CEF, CMF, AC, AC-T) in the treatment of breast cancer. Cyclophosphamide itself is a pro-drug which undergoes a a 4-hydroxylation step in the liver and then spontaneous ring opening to form phosphoramide mustard and acrolein. While the former is primarily responsible for gonadotoxic effects, the latter causes urinary toxicity. The formation of guanosine adducts that prevent DNA replication and damage to mitochondria and other organelles within cells are the primary mechanisms of the cyclophosphamide-induced cellular cytotoxicity [7, 16]. Cyclophosphamide is metabolically activated by CYP2B6 enzyme and induces cellular death by forming DNA adducts, in particular, the interstrand DNA crosslink, through formation of the active metabolite, phosphoramide mustard. The drug primarily alkylates the N-7 position of guanine and forms 67% phosphotriester monoadducts, 26% N-7-guanine monoadducts, and 6.7% N-7-guanine-N-7 guanine interstrand crosslinks. Even though interstrand crosslinks comprise only 5–10% of all adducts formed by alkylating agents, these adducts are the most physiologically relevant because of their ability to block DNA replication and their association with cytotoxicity [17, 18]. In human fetal ovaries xenografted to immune deficient mice cyclophosphamide caused massive apoptosis of oocytes and granulosa cells of primordial follicles starting as early as 24 h after administration of the drug as an in-vivo real time histomorphometric evidence of the gonadotoxicity of the drug [5].

Chemotherapy drugs used in treatment of breast cancer have ovarian toxicity profiles that vary according to their cell cycle-specificity toxicity (Figs. 1 and 2). For instance, the alkylating agents and anthracyclines (non-specific cell-cycle agents) are capable of impacting cell at every stages of cell cycle. Therefore these drugs induce more widespread damage to the ovary. By contrast, cell-cycle-specific agents (e.g., 5-fluorouracil and methotrexate) could be more likely to cause damage to the preantral and antral follicles than primordial follicles due to their higher mitotic rate and metabolic demand. Although there is still no direct in-vivo histomorphometric evidence in human ovary, the evidence supporting this notion came from a mouse study, which demonstrated that multidose 5-FU treatment resulted in dramatic and progressive atresia of growing follicles and a profound decrease in ovarian volume due to reduced corpus luteum counts. Primordial follicle numbers were not affected and therefore, 5-FU is unlikely to cause permanent infertility when administered to women of pre or reproductive age. Furthermore, this study suggests that depletion of the growing follicle population is insufficient to stimulate follicle activation and primordial follicle depletion [19].

### The Risk of Ovarian Failure and Amenorrhea after Receiving Conventional Chemotherapy Drugs for Breast Cancer

#### Alkylating Regimens

CMF (cyclophosphamide, methotrexate, and 5-fluorouracil) is one of the most commonly employed chemotherapy regimen of alkylating category. The incidence of amenorrhea following receiving CMF regime widely changes from 0% to 97% in premenopusal women [20, 21] due to heterogeneity in patients’ age, duration of treatment and follow-up [4]. Younger patients are more likely to have higher ovarian reserve and retain more residual ovarian function after completion of chemotherapy. As an example, Bines et al. reported that ovarian failure incidence after the CMF regime is lower in patients younger than 40 (respectively, 40% vs. 76%) [22]. The amenorrhea risk is higher in older patients because of age-related decline in their ovarian reserve. In another series, none of the 25 patients younger than age 35 developed amenorrhea after six courses of CMF, whereas 33.3% and 80% of the patients at ages between 36 and 40 and 41–45, respectively, developed amenorrhea after receiving the same regimen [20]. Moreover, temporary amenorrhea (hazard ratio = 1,96) and CMF X 6–7 (hazard ratio = 2.03) are related to premature menopause [23].

CEF or FEC (Cyclophosphamide, Epirubicin, and 5-Fluorouracil) is another alkylating regimen used for breast cancer treatment. One study showed that the risk of amenorrhea after CEF appears to be significantly higher compared to CMF (76% vs. 64%; respectively, relative risk, 1.2; 95% CI, 1.0 to 1.3) [24]. However, other studies found comparable rates of amenorrhea after CMF and CEF regimens [25–28]. The risk of amenorrhea rate after CEF calculated based on the eight studies including 1190 breast cancer patients to be 51% after six courses at 12 months of follow-up [24–31]. In the multicentre PACS 01 trial, Berliere et al. indicated comparable amenorrhea rates between patients treated with six cycles of FEC (6FEC) vs. three cycles of FEC followed by three cycles of docetaxel (3FEC/3D) at the end of chemotherapy (93% vs. 92.8%). On the other hand, in one year period, returning premenopausal hormone levels and recovered menses were more frequent in the 3FEC/3D arm than in the 6FEC arm (respectively; 43% vs. 29%, 35.5% vs. 23.7%) [29]. Zhou et al. showed different amenorrhea rates between various chemotherapy protocols; 44.87% for the FEC and 30.30% for the TE (docetaxel-epirubicin), and 23.08% for the NE (navelbine-epirubicin). Furthermore, although significant differences were not found between the FEC and TE groups, the amenorrhea rate was significantly higher in the FEC compared to the NE regimen [30].

#### Anthracyclines

Anthracyclines (doxorubicin, daunorubicin, epirubicin and idarubicin) exert antineoplastic effect by inhibiting the nuclear topoisomerase-II enzyme, and promoting chromosome disentanglement. Therefore, topoisomerase-II DNA complex formation is prevented, leading to the accumulation of DNA fragments and eventual death of the cell [16]. Cell viability decreases along with mitochondrial damage and free radical formation. Gonadal effects of doxorubicin are more widely investigated using several animal models [16, 32] and two human studies [33, 34]. These studies show that doxorubicin reduces ovulation rate, blastocyst formation, and reproductive life span. It also causes damage to ovarian stroma and vascular structures by inducing toxicity to both oocyte and granulosa cells of the primordial and early growing follicles. A recent animal study on mouse ovary demonstrated that doxocycline, cisplatin and taxanes can induce the apoptosis of ovarian granulosa cells (GCs), likely resulting from excessive reactive oxygen species (ROS) production-induced oxidative damage and impaired cellular anti-oxidative capacity [35].

Anthracycline-based regimens are less detrimental to the ovaries than alkylating regimens because of the lower total dosage of cyclophosphamide. For instance, 74% of patients treated with CMF developed amenorrhea in contrast to 42% receiving AC regimen [adriamycin (doxorubicin) and cyclophosphamide] [36]. Similar results was obtained in another study, which demonstrated that amenorrhea rates in patients treated with AC and CMF regimens were 53% and 83%, respectively [37]. Hortobagyi et al. reported that no patients under 30 years of age experienced menstrual abnormalities, whereas 96% of those 40–49 years of age developed amenorrhea, which was permanent for most women over 40 but was reversible for 50% of patients under 40 years of age [38]. The subsequent studies confirmed that the AC regimen did not cause amenorrhea in patients younger than 35 during two years of follow-up [39] or caused amenorrhea only in 13% of cases younger than age 40 [40]. Kil WJ et al. studied menstrual changes in breast cancer patients under 35 years of age in a retrospective study. They found similar amenorrhea occurrences (25 (31.3%) in those treated with CMF, and 34 (42.5%) in anthracycline-based regimens (AD) (*p* = 0.142)) and menstruation resumed in 83.1% of patients, 80% of those treated with CMF, and 85.3% with AD. The study indicates that CMF and AD protocols have similar amenorrhea or recovery rates within 54 months of the follow-up period [41]. It appears that while the risk of permanent ovarian failure is low in anthracycline regimens compared to alkylating ones in young patients, caution should still be exercised when treatingpatients older than age 35 with AC because the risk is elevated.

#### Taxanes

Taxanes (paclitaxel and docetaxel) exert antineoplastic effects by stabilizing mitotic spindles by attaching to tubulin within existing microtubules [42, 43]. As a result, the normal microtubule breakdown (depolymerization) during cell division is impaired. Activation of the mitotic checkpoint induces reversion to the G-phase of the cell or apoptosis. (mitotic arrest). Besides, taxanes can prevent maturation of the oocyte as they prevent depolymerization of the meiotic spindles during meiosis I and II [44]. The impact of taxanes on primordial follicle pool is unknown in human and data on mouse ovary is inconsistent [45]. Taxanes are used in combination with other chemotherapy drugs, AC (doxorubicin, cyclophosphamide), EC (epirubicin, cyclophosphamide), FAC (fluorouracil, doxorubicin, cyclophosphamide) and FEC (fluorouracil, epirubicin, cyclophosphamide). Therefore it is difficult to determine its individual gonadotoxic potential. Several studies compared the amenorrhea rates between these protocols with and without taxanes. Tham YL et al. demonstrated that the rate was higher in AC + T than AC alone regimen (64% vs. 55%) [46]. By contrast, Reh et al. found no significant difference in amenorrhea rates within six months of follow-up after chemotherapy (ACT group, 29%; AC group, 41.7%). However, within the second follow-up period (28 months after chemotherapy; mean), they found higher amenorrhea rates in the ACT patients and suggested that gonadotoxicity of paclitaxel may manifest itself at more extended follow-up periods (35.7% in the ACT group, vs. 9.1% in the AC group) [47]. Another study demostrated AC and AC + T regimens exhibited similar amenorrhea rates after two years, (66.7% vs. 73.3%, respectively) [48]. Okanami et al. monitored the long-term effects of taxane on the ovary and showed that even though the amenorrhea rates during chemotherapy are significantly higher in the anthracycline+taxane arm than in the anthracycline (93.9% vs. 73.6%, *p* < 0.05), persistent amenorrhea rates were similar, 24.5% vs. 11.8% (*p* > 0.05) after three years of follow-up. The authors concluded that patients’ age at diagnosis (≤ 35 vs. ≥36) was significantly and independently associated with chemotherapy-related amenorrhea within long-term follow-up. In addition, patients in the AC + T group had longer times to resume menstruation than in AC (342 vs. 92 days) [49]. Comparison of AC, AC + T and CMF regimens in 466 premenopausal breast cancer patients forthe likelihood of resumption of menstrual bleeding after six months of amenorrhea differed significantly by treatment protocols (*P* = 0.002; 68% with AC, 57% with AC + T, and 23% with CMF). Nevertheless, no significant difference was reported between the chemotherapy protocols for recovery after 12 months of amenorrhea. And after two years Patients treated with CMF mainly continued to have amenorrhea (84%), followed by AC + T and AC (68%, 58%) [39]. However, another studies did not show a significant increase in the risk of amenorrhea after the sequential addition of a taxane to standard adjuvant anthracycline-based chemotherapy compared to historic controls [50] or demonstrated that the likelihood of remaining amenorrheic was not statistically different in patients who received AC-T versus AC at 33 months of follow-up (OR, 1.59; 95% CI, 0.8–3.2) [40]. A meta-analysis of 8 studies with 2124 patients found that the adjusted OR for chemotherapy-induced amenorrhea in patients receiving taxanes vs. no-taxanes combined with anthracyclins is 1.45 (0.94–2.23), suggesting a weak correlation between taxanes and the occurrence of amenorrhea [51].

Docetaxel is a semi-synthetic analog of paclitaxel. So far several clinical studies analyzed the impact of docetaxel on ovarian function in premenopuasl women with breast cancer. The PACS01 trial showed comparable amenorrhea rates between FEC vs. 3FEC/3D (93% vs. 92.8%). However, more patients in the 3FEC/3D arm recovered menses (35.5% vs. 23.7%, *p* < 0.05) within one year period. The study also showed that patients under age 40 treated with taxane-containing regimens had an increased incidence of reversible amenorrhea (20.5 vs. 10.5%, *P* = 0.025). In contrast, the incidence of amenorrhea was high in both groups for women > 40 years of age [29]. In the BCIRG 01 trial, amenorrhea rates were lower in the FAC arm than TAC (docetaxel, doxorubicin, and cyclophosphamide) regimen (32.8% vs. 51.4%, respectively) [52]. The NSABP B-30 trial assessed the likelihood of resumption of menses according to age of the patients, which were 3.2% for women > 50 years, 10.9% for women 40–50, 45.3% for patients under 40 years of age in premenopausal women received on the AC-T regimen (doxorubicin-and-cyclophosphamide-followed-by-docetaxel) [53]. The investigators also found that those treated with tamoxifen were more likely to become amenorrheic (*p* = 0.003). Prolonged amenorrhea was associated with a regimen that contains doxorubicin, cyclophosphamide, and docetaxel, and was age dependent and impacted by tamoxifen use [53]. Also, a substudy of this trial with 2445 patients found significant differences in amenorrhea rates among those protocols (69.8% for AC + T, 57.7% for TAC, and 37.9% for AT (*P* < 0.001)) [54].

A prospective cohort study showed that the amenorrhea rates were 90.2% for the TX/AC (docetaxel and capecitabine/doxorubicin and cyclophosphamide), 73.5%, for the AC-T was and 72.1% for the FAC regimen in one year, and were 66.7%, 73.3%, and 58.9% respectively, in 3 years. In addition, multivariate analysis showed that the amenorrhea rates were significantly associated with age (*P* < 0.001) and taxane use (*P* = 0.002) in the 1-year follow-up [48].

Nafaji et al. analyzed 226 breast cancer patients and found that the risk for amenorrhea was higher in taxane based protocols (52.5%, 66.7%, and 78.7% for CMF, anthracycline, and anthracycline-taxane regimens respectively). The two most significant factors for the development of amenorrhea were anthracycline-taxane-based protocols (OR: 4.059; 95% CI: 1.6–9.8) and over 40 years of age (OR, 3.5; 95% CI, 1.9–6.6) [55]. Comparison of FEC with sequential ECT, FEC-T, and concurrent ECT regimens on 165 breast cancer patients found that regimens including taxane did not increase the rate of amenorrhea compared to FEC regimens (P.0.05 for all) [56]. Another study comparing CAF (tegafur + pirarubicin + ifosfamide) with DTC (docetaxel + pirarubicin + ifosfamide) in 164 women found that DTC carries a significantly higher risk of premature ovarian failure than the CAF regimen [57].

### The Impact of Tamoxifen and Targeted Drugs on Ovarian Function

#### Tamoxifen

Tamoxifen acts as a selective estrogen receptors modulator and exerts its antiestrogenic actions by competing with estrogen at its receptor site. The drug has been used as a first-line treatment of estrogen receptor-positive breast cancer for decades [58]. Data on the association between tamoxifen use and amenorrhea is still controversial. While several studies found no significant differences between patients who were treated with tamoxifen or not [28, 50, 59]. Prospective follow-up of the patients in the IBCSG (International Breast Cancer Study Group) trial 13–93 showed comparable amenorrhea rates in women who did and did not receive tamoxifen after chemotherapy (88% vs. 84%) [60]. On the other hand, other studies demonstrated higher amenorrhea rates with tamoxifen treatment. For instance, in a study of 466 premenopausal breast cancer patients who were administered AC, ACT and CMF protocols with and without tamoxifen and followed up at 6-month intervals, the odds of experiencing 6, 12 and 24-month amenorrhea periods were 2.72 (95% CI, 1.64–4.50), 2.96 (95% CI, 1.68–5.24). Women treated with tamoxifen had 2.54 times (95% CI: 1.32–4.88) higher risk for a 24-month amenorrhea period than those not treated [39]. Abusief et al. found in retrospective analysis that patients over forty had two times higher risk of staying amenorrheic (OR, 2.51; 95% CI, 1.20–5.2). Amenorrhea was associated significantly with TAM use and age at diagnosis [40]. Another retrospective study in 250 premenopausal women with breast cancer yielded similar findings. The menstrual abnormalities were more frequently observed in the patients treated with tamoxifen (amenorrhea: 22% vs. 3%, *p* < 0.001) without any difference at menopause age [61]. Two different meta-analyses have demonstrated that tamoxifen significantly increased the incidence of amenorrhea in premenopausal breast cancer patients [51, 62]. The precise mechanism underlying tamoxifen-related amenorrhea is yet to be fully elucidated, but evidence suggests a functional rather than cytotoxic etiology. Given that anti-Müllerian hormone levels often remain comparable between patients receiving anti-estrogenic versus non-anti-estrogenic therapy, we hypothesize that tamoxifen primarily exerts a central inhibitory effect on the hypothalamic-pituitary-gonadal (HPG) axis via its estrogen receptor modulation, rather than causing direct ovarian toxicity. Consequently, tamoxifen might be responsible for amenorrhea but not for ovarian failure [63]. A recent study demonstrated that ovarian reserve significantly decreased in the AC and CMF protocols compared to the tamoxifen-only treatment (*p* < 0.0001 for both AC vs. tamoxifen and CMF vs. tamoxifen). Furthermore, substantially diminished anti-Müllerian hormone levels were seen and comparable at the AC and CMF regimens (*p* = 0.53). Lastly, recuperation of anti-Müllerian hormone levels after 12 to 18 or 18 to 24 months after these protocols was insignificant. However, patients treated with the tamoxifen-only protocol showed minor changes in the age-adjusted anti-Müllerian hormone levels, and adjuvant tamoxifen treatment after the AC protocol did not impact anti-Müllerian hormone recovery [64]. Tamoxifen may be used in an ovulation induction as clomiphene citrate is also used for patients with anovulatory infertility issues. In addition, it can be used in breast cancer patients who underwent IVF treatment based on its dual-action [65, 66].

#### Trastuzumab and Lapatinib

Trastuzumab is an anti-HER2 monoclonal antibody, and lapatinib is a tyrosine kinase inhibitor. Both drugs are approved in the treatment of human epidermal growth factor receptor 2 (HER2/neu receptor) positive breast cancers, which constitutes 25% of the tumors [67, 68]. Epidermal growth factor is normally expessed by human ovary and play pivotal roles in follicle growth, ovulation and oocyte maturation [69]. Although still limited available evidence do not show any detrimental effect of inhibition of or antagonizing EGF receptor with these drugs on ovarian function and reserve in premenopausal women with breast cancer. In a cross-sectional study of 100 premenopausal breast cancer patients of whom 25 received trastuzumab, the investigators observed an association on multivariate analysis between trastuzumab exposure and higher serum anti-Müllerian hormone levels in the survivors with regular cycles [70]. Other studies obtained similar results. Abusief et al. found that the risk of amennorrhea development did not actually increase in patients treated with either 12 (short-term) or 52 doses (long-term) of the trastuzumab regimen. Furthermore, based on the multivariate analysis of 431 patients with a 33-month median follow-up period (range, 6–114 months), regarding the odds of remaining amenorrheic, no difference was found between the AC-T and the AC protocols ((OR; 1.59; 95% CI, 0.8–3.2)), and dose-dense versus every three-week treatment (OR, 0.56; 95% CI, 0.25–1.3), or AC-T + trastuzumab (OR, 0.6;95% CI, 0.22–1.61). On the other hand, tamoxifen usage and age at the diagnosis were significantly associated with the amenorrhea [40]. The NRG Oncology/NSABP B-47 menstrual history study showed similar rates of amenorrhea with or without the addition of trastuzumab, with 84% of pre-and perimenopausal women experiencing amenorrhea in the trastuzumab arm compared with 86.3% in the non-trastuzumab group at 12 months [71].

In the single-arm phase 2 APT (adjuvant paclitaxel-trastuzumab) trial, Ruddy et al. demonstrated that the amenorrhea rate was 28% (95% CI: 18–41%) and concluded that this rate appears to be lower than anticipated rates at the traditional alkylating chemotherapy protocols [72]. In a multi-center, open-labeled, phase 3 ALTTO (Adjuvant Lapatinib and/or Trastuzumab Treatment Optimization; BIG 2–06) trial, 2,862 HER2 positive premenopausal breast cancer patients were randomized to four groups (lapatinib alone, trastuzumab alone, their sequence or their combination). The rates of treatment-induced amenorrhea did not show a difference in the four arms, being 72.6%, 74.0%, 72.1%, and 74.8%, respectively. However, since the study group did not include patients without anti-HER2 therapies, they could not address the potential gonadotoxic effect of these therapies beyond the chemotherapy [73, 74]. Lapatinib inhibits meiotic maturation of porcine oocytes in vitro [75]. Given that fact both trastuzumab and lapatinib block EGFR2 signaling, which is required for cumulus expansion and completion ovulation and meiotic maturation of the oocyte it would not be surprising to see the same effect in human ovary as well.

More recently, newer anti-HER2 antibody–drug conjugates (ADCs), including ado-trastuzumab emtansine (T-DM1) and trastuzumab deruxtecan (T-DXd), have expanded therapeutic options for HER2-positive breast cancer. Unlike naked transtuzumab, emtansine and deruxtecan exert their cytotoxic effects directly to HER2-expressing cells. In the ATEMPT trial, which compared adjuvant T-DM1 with paclitaxel plus trastuzumab in early stage HER2-positive breast cancer patients, chemotherapy-related amenorrhea at 18 months was reported to be less frequent in the T-DM1 arm, suggesting a potentially more favorable ovarian safety profile compared with conventional chemotherapy-based regimens [76]. Nevertheless, amenorrhea may not be sufficient enough to fully capture the overall impact on ovarian function. Future studies incorporating biochemical or ultrasonographic markers such as anti-Müllerian hormone or antral follicle count may provide a more comprehensive assessment of ovarian effects. For trastuzumab deruxtecan, no direct clinical data on ovarian reserve or fertility outcomes in breast cancer patients are currently available. In DESTINY-PanTumor02 trial, trastuzumab deruxtecan showed clinically relevant antitumor activity in previously treated HER2-expressing solid tumors, with a safety profile consistent with prior reports, although gonadotoxic effects were not evaluated [77].

#### Poly ADP-ribose (PARP) Inhibitors

PARP inhibitors (PARPi) are used in the treatment of breast cancer because BRCA mutant tumors have defects in DNA repair mechanisms [homologous repair deficiency (HRD)], therefore, have increased sensitivity to both DNA-damaging agents and poly (ADP-ribose) polymerase (PARP) inhibitors [78, 79]. Preclinical studies demonstrated that PARPi significantly reduced primordial follicle reserve in mouse ovary without affecting growing follicle fractions. It has been also been shown in mouse model that PARPi may also impair granulosa cells function [80–82]. As a preliminary report we have shown using an ex-vivo human ovarian explant culture model that the primordial follicle reserve and steroidogenic activity of the tissue samples remained stable up to 96 h in culture. Similar results were obtained in the granulosa cells, which retained their viability and steroidogenic functions after exposure to the PARP inhibitors. Real-time impedance-based cell proliferation assay with xCelligence system revealed that granulosa cells continued to proliferate normally and reached log phase in the presence of PARP inhibitors without any discernible cytotoxic effect. In immunoblot analysis, the expression of steroidogenic enzymes (StAR, aromatase, 3B-HSD and 17B-HSD) was comparable to control cells and cleaved caspase-3 expression was absent in the granulosa cells exposed to PARP inhibitors [83]. While these findings provide some reassurance more studies are needed to better define their gonadotoxic potentials if any.

#### Cyclin Dependent Kinase Inhibitors

Cyclin-dependent kinase 4/6 (CDK4/6) inhibitors interrupts mitosis by blocking the transition from the G1 to the S phase, through phosphorylation of the retinoblastoma protein 1. Palbociclib, ribociclib, and abemaciclib are all approved for treating advanced hormone receptor-positive, HER2-negative breast cancer [84]. Preclinical studies in rat ovary treated with high doses of palbociclib showed no detrimental effect on fertility [85]. It was demonstrated that in vitro treatment of human mitotic granulosa cells with CDK4/6 inhibitors caused a dose-dependent accumulation of the cells at G_0_/G_1_, S phase and G_2_/M transition on flow cytometric analysis and inhibited their mitotic proliferation. However, the viability, the expression of steroidogenic enzymes StAR, aromatase and estrogen production of the granulosa cells were not affected by these drugs compared to control cells [86].

#### Immune Check Point Inhibitors (ICIs)

Immune checkpoint inhibitors (ICIs), such as pembrolizumab are used in the treatment of early-stage triple-negative breast cancer [87]. ICIs drugs are designed to block the inhibitory signals of tumor cells, which eventually allows immune cells to attack and destroy tumor cells more effectively. The targeted molecules on tumor cells are cytotoxic T lymphocyte-associated molecule-4 (CTLA-4), programmed cell death receptor-1 (PD-1), and programmed cell death ligand-1 (PD-L1) [88]. However, this breach of immune tolerance can lead to immune-related adverse events (irAEs) in healthy tissues. The prevailing pathophysiological hypothesis for ICI-induced gonadotoxicity is “autoimmune oophoritis.” Since the ovary is not an immune-privileged site, the systemic activation of T-cells may lead to the recognition of ovarian self-antigens. Preclinical models support this; mice treated with ICIs exhibit lymphocytic infiltration (CD4 + and CD8 + T cells) into the ovarian cortex, increased broad-spectrum inflammation (TNF-α, IFN-γ), and a consequent reduction in both the primordial follicle pool and corpus luteum formation [89].

Clinically, evidence is accumulating from both trials and real-world data. While specific fertility data from breast cancer trials remain limited, the ECOG-ACRIN E1609 phase III trial in melanoma provided pivotal prospective evidence, showing a distinct trend of reduced ovarian reserve (declining anti-Müllerian hormone and rising FSH) in women treated with ipilimumab and nivolumab [90–92]. (Table 1). Moreover, ICIs are also rexognized for their other endocrinological and immune mediated side effects such as hypophysitis and impairement of hypothalamic-pituitary-gonadal axis. Using NIH FEARS data base we have very recently demonstrated that spermatogenesis abnormality in males and genital tract fistula in females had the strongest association with the use of ICI inhibitor drugs. PD-1 inhibitors pose greater risk than CTLA-4 inhibitors (OR = 1.65 [1.05–2.79], *p* = 0.045) [96]. This suggests that the spectrum of ICI-induced reproductive toxicity extends beyond functional ovarian reserve depletion to include severe structural tissue damage, likely driven by profound immune-mediated mucosal inflammation and necrosis. Based on the available evidence counseling for fertility protection is critically required for young women of reproductive age who are scheduled to receive ICI drugs for breast cancer.

**Table 1:** This table summarizes different classes of targeted therapies, their representative agents and mechanisms of action with corresponding evidence on ovarian effects from human and animal studies. While some agents such as PARP inhibitors and CDK4/6 inhibitors show measurable alterations in granulosa cell function and follicle pools in preclinical models, others (e.g., trastuzumab) appear to have minimal or no adverse impact on ovarian reserve. Data on immune-checkpoint inhibitors and tyrosine kinase inhibitors suggest potential immune-mediated or meiotic effects on ovarian tissue, although further studies are warranted. *ADP* adenosine diphosphate, *PARP* Poly (ADP-ribose) polymerase, *DNA* deoxyribonucleic acid, *BRCA* breast cancer susceptibility gene, *CDK4/6* Cyclin-dependent kinase 4/6, *CTLA-4* cytotoxic T lymphocyte-associated molecule-4, *PD-1* programmed cell death receptor-1, *PD-L1* programmed cell death ligand-1, *HER2* human epidermal growth factor receptor 2

Group	Name	Mechanism of action	Human	Animal
Poly (ADP-ribose) polymerase (PARP) inhibitors	Olaparib, Niraparib, Rucaparib	Inhibits DNA repair in BRCA mutation carriers	No effect on in-vitro mitosis and steroidogenic function of granulosa cells [83]	Depletion of primordial follicle pool, granulosa cell dysfunction in mouse ovary. [80–82].
Cyclin-dependent kinase 4/6 (CDK4/6) inhibitors	Palbociclib, Ribociclib, Abemaciclib	Interrupt mitosis by blocking the transition from the G1 to the S phase, through phosphorylation of the retinoblastoma protein 1	Inhibition of mitotic proliferation of human granulosa cells and delay in their cell cycle progression without any discernible effect on viability and steroidogenic function [93]	No detrimental effect of high dose CDKis on rat ovary [85]
Immune -check point inhibitors	Pembrolizumab	Enhancement of tumor killing by blocking cytotoxic T lymphocyte-associated molecule-4 (CTLA-4), programmed cell death receptor-1 (PD-1), and programmed cell death ligand-1 (PD-L1)	Immune mediated destruction of follicles and reduction in ovarian reserve [90]	Immune mediated destruction of follicles and reduction in ovarian reserve [89]
Anti-HER2 monoclonal antibody	Trastuzumab	Inhibition of growth of human epidermal growth factor receptor 2 (HER2/neu receptor) positive breast cancers	No detrimental effect on ovarian function and reserve [71]	May mitigate chemotherapy-induced ovarian toxicity via vascular mechanisms; no evidence of direct gonadotoxicity. [94]
Tyrosine kinase inhibitor	Lapatinib	Inhibition of growth of human epidermal growth factor receptor 2 (HER2/neu receptor) positive breast cancers	Possible detrimental effect on meiotic maturation of oocytes [75]	Inhibits meiotic maturation of porcine oocytes [75, 95]

### Role of Breast Cancer Susceptibility Genes on Ovarian Reserve and Function before and after Chemotherapy

An important topic requiring further investigation and clarification is the possible increase in treatment-induced gonadotoxicity among young breast cancer patients carrying mutations in breast cancer susceptibility genes. These susceptibility genes includes, but are not limited to, BRCA1, BRCA2, ATM, CHEK2, PALB2, BARD1, RAD51C, RAD51D, and TP53 [97]. However, most studies have primarily focused on breast cancer patients with germline deleterious BRCA1 or BRCA2 mutations, as this subgroup represents the majority of hereditary breast cancer cases.

Chemotherapeutic agents affect human ovaries directly by inducing DNA damage and indirectly by impairing stromal cells and vascular structure [5]. Since BRCA plays a crucial role in repairing chemotherapy-induced DNA damage it can be hypothesized that treatment-induced gonadotoxicity is more pronounced in breast cancer patients carrying BRCA mutations compared to non-carriers. However, the currently available data is limited and controversial. A study by Valentini et al. reported no significant difference in the incidence of chemotherapy-induced amenorrhea (≥ 2 years without menses) between BRCA-mutated and BRCA-negative premenopausal breast cancer patients receiving chemotherapy (35.6% vs. 49%; *P* = 0.18). Notably, chemotherapy-induced amenorrhea was significantly more common in BRCA2 carriers than in BRCA1 carriers (46.8% vs. 32.7%; *P* < 0.001) [98]. Theoretically, mutations located in regions critical for protein function (e.g., DNA binding domains) could result in a more profound compromise of homologous recombination repair, thereby rendering oocytes more susceptible to DNA-damaging agents and accelerating the depletion of the ovarian reserve. While the concept of “ovarian cluster regions” (well-defined for cancer risk) has not yet been fully mapped to fertility outcomes, these findings emphasize the importance of moving beyond binary “carrier vs. non-carrier” analyses toward more granular genotype-phenotype correlations in future fertility preservation research. A single-center prospective study with a small cohort revealed no significant difference in anti-Müllerian hormone levels at baseline (1.94 vs. 1.66 µg/L; *P* = 0.53), 1 year (0.09 vs. 0.06 µg/L; *P* = 0.39), and 3 years (0.25 vs. 0.16 µg/L; *P* = 0.43) after chemotherapy between BRCA-mutated and BRCA-negative young (≤40 years) early breast cancer patients [99]. On the other side BRCSA mutations have been shown to be independent risk factor associated with baseline reduced ovarian reserve and increased risk of postchemotherapy amenorrhea [100–102]. No information available in literature regarding the effects of other breast cancer susceptibility genes in baseline ovarian reserve before and after chemotherapy in breast cancer patients.

## Conclusion

Conventional and targeted breast-cancer treatments can affect the ovaries in four main ways: direct DNA injury to oocytes and granulosa cells, vascular and stromal compromise, premature follicle activation (“burn-out”) and drug-specific cell-cycle arrest. Severity of the damage depends on the drugs used. Alkylating combinations such as CMF and CEF remain the most gonadotoxic, anthracycline-only or sequential taxane regimens carry an intermediate risk and weekly paclitaxel or anti-HER2 therapy appear to be least harmful. Age and starting ovarian reserve has the biggest impact, yet germ-line DNA-repair defects, especially BRCA1/2 variants, amplify vulnerability even before treatment begins. Early signals suggest ATM, CHEK2, PALB2, and other genes may pose similar risks, so genetics should be part of every counseling session.

Newer targeted drugs raise fresh questions. Laboratory studies revealed that PARP inhibitors can reduce the primordial follicle pool, and real-world pharmacovigilance link PD-1 blockade to hormone problems. By contrast, early studies of CDK4/6 inhibitors and anti-HER2 agents haven’t shown major fertility red flags, though follow-up is still short. Consequently, fertility counseling should be risk-stratified based on the specific drug class and the patient’s genetic background. For patients scheduled for high-risk alkylating regimens (e.g., cyclophosphamide-based), particularly BRCA mutation carriers who may already possess a compromised ovarian reserve, immediate referral for oocyte or embryo cryopreservation is mandatory prior to the initiation of therapy. In contrast, for patients prescribed targeted agents such as PARP inhibitors or Immune Checkpoint Inhibitors (ICIs), counseling faces a “grey area.” Although human data regarding long-term gonadotoxicity is limited, preclinical evidence of primordial follicle depletion suggests these drugs are not benign. Therefore, clinicians should adopt a precautionary approach: fertility preservation should be offered not only to insure against potential drug-induced injury but also to mitigate the natural ovarian aging that occurs during prolonged maintenance therapy. Finally, for patients on lower-risk regimens (e.g., anti-HER2 antibodies), counseling should focus on the duration of the proposed treatment and the impact of age-related fertility decline, rather than direct drug cytotoxicity. Fast-track egg or embryo freezing remains the gold standard for preservation, while GnRH agonists may offer concurrent ovarian protection but should not replace cryopreservation methods. Building a standard pretreatment that combines reliable ovarian-reserve markers (anti-Müllerian hormone, antral-follicle count) with germ-line genetic testing is crucial. This way, every patient can get a clear, individualized risk estimate. However, it must be noted that the interpretation of the current literature is often limited by significant heterogeneity across studies, particularly regarding varying definitions of chemotherapy-induced amenorrhea and differing follow-up durations. Moreover; truly long-term evaluation of ovarian function recovery remains limited. Available data largely rely on menstrual status or age at menopause as endpoints, rather than serial assessment of ovarian reserve markers. As a result, these studies cannot adequately delineate the temporal trajectory of ovarian recovery of late functional decline after cancer treatment. Gathering reproductive outcomes from further studies, such as, amenorrhea rates, biochemical POI and live births with standardized endpoints will sharpen our understanding of true gonadotoxicity and let clinicians give patients genuinely informed choices about both cancer control and future fertility projections. 

## Abbreviations

3B-HSD: 3-beta-hydroxysteroid dehydrogenase
AC: Adriamycin (doxorubicin) + cyclophosphamide
AC-T/ACT: AC followed by a taxane (paclitaxel or docetaxel)
AC + T: Adriamycin + cyclophosphamide with a concurrent or sequential taxane
ADCs: Antibody–drug conjugates
Akt (PKB): Protein kinase B
AMH: Anti-Müllerian hormone
APT: Adjuvant paclitaxel–trastuzumab regimen
ATM: Ataxia telangiectasia mutated (serine/threonine protein kinase)
BARD1: BRCA1-associated RING domain 1
BRCA1: Breast-cancer susceptibility gene/protein type 1
BRCA2: Breast-cancer susceptibility gene/protein type 2
CAF: Tegafur + pirarubicin + ifosfamide
CEF: Cyclophosphamide + epirubicin + 5-fluorouracil
CHEK2: Checkpoint kinase 2
CMF: Cyclophosphamide + methotrexate + 5-fluorouracil
CTLA-4: Cytotoxic T-lymphocyte-associated protein 4
CYP2B6: Cytochrome P450 family 2 subfamily B member 6
DNA: Deoxyribonucleic acid
DTC: Docetaxel + pirarubicin + ifosfamide
ECT: Epirubicin + cyclophosphamide + taxane (docetaxel)
EGF/EGFR: Epidermal growth-factor/epidermal growth-factor receptor
FAC: 5-Fluorouracil + adriamycin (doxorubicin) + cyclophosphamide
FEC: Epirubicin + cyclophosphamide
FEC-T: FEC followed by docetaxel
FOXO3a: Forkhead box O3a
GC: Granulosa cell
HER2: Human epidermal growth-factor receptor 2
HPG: Hypothalamic-pituitary-gonadal
HRD: Homologous recombination deficiency
ICIs: Immune check-point inhibitors
IFN-γ: Interferon-gamma
irAEs: Immune-related adverse events
IVF: In vitro fertilization
mTOR: Mechanistic (Mammalian) target of rapamycin
PALB2: Partner and localizer of BRCA2
PARP/PARPi: Poly (ADP-ribose) polymerase/poly (ADP-ribose) polymerase inhibitors
PD-1: Programmed cell-death protein 1
PI3K: Phosphatidylinositol-3-kinase
POI: Premature ovarian insufficiency
RAD51C: RAD51 paralog C
RAD51D: RAD51 paralog D
RI: Resistive index
ROS: Reactive oxygen species
StAR: Steroidogenic acute regulatory protein
TAC: Docetaxel + adriamycin (doxorubicin) + cyclophosphamide
TAM: Tamoxifen
T-DM1: Ado-trastuzumab emtansine (trastuzumab-DM1 conjugate)
T-DXd: Trastuzumab deruxtecan
TNF-α: Tumor necrosis factor-alpha
TE: Docetaxel + epirubicin
TP53: Tumor protein p53
TX/AC: Docetaxel + capecitabine, then adriamycin + cyclophosphamide
VEGF: Vascular endothelial growth factor
